# A transcriptomic atlas of aged human microglia

**DOI:** 10.1038/s41467-018-02926-5

**Published:** 2018-02-07

**Authors:** Marta Olah, Ellis Patrick, Alexandra-Chloe Villani, Jishu Xu, Charles C. White, Katie J. Ryan, Paul Piehowski, Alifiya Kapasi, Parham Nejad, Maria Cimpean, Sarah Connor, Christina J. Yung, Michael Frangieh, Allison McHenry, Wassim Elyaman, Vlad Petyuk, Julie A. Schneider, David A. Bennett, Philip L. De Jager, Elizabeth M. Bradshaw

**Affiliations:** 1 Center for Translational & Computational Neuroimmunology, Department of Neurology, Columbia University Medical Center, New York City, NY 10032 USA; 2 Program in Medical and Population Genetics, Broad Institute, Cambridge, MA 02142 USA; 3 School of Mathematics and Statistics, The University of Sydney, Sydney, New South Wales 2006 Australia; 4 Massachusetts General Hospital, Boston, MA 02114 USA; 5 Ann Romney Center for Neurologic Diseases, Brigham and Women’s Hospital, Harvard Medical School, Boston, MA 02115 USA; 6 Pacific Northwest National Laboratory, Richland, WA 99354 USA; 7 Rush Alzheimer’s Disease Center, Rush University Medical Center, Chicago, IL 60612 USA

## Abstract

With a rapidly aging global human population, finding a cure for late onset neurodegenerative diseases has become an urgent enterprise. However, these efforts are hindered by the lack of understanding of what constitutes the phenotype of aged human microglia—the cell type that has been strongly implicated by genetic studies in the pathogenesis of age-related neurodegenerative disease. Here, we establish the set of genes that is preferentially expressed by microglia in the aged human brain. This HuMi_Aged gene set captures a unique phenotype, which we confirm at the protein level. Furthermore, we find this gene set to be enriched in susceptibility genes for Alzheimer’s disease and multiple sclerosis, to be increased with advancing age, and to be reduced by the protective *APOEε2* haplotype*. APOEε4* has no effect. These findings confirm the existence of an aging-related microglial phenotype in the aged human brain and its involvement in the pathological processes associated with brain aging.

## Abstract (web summary)

Aging is associated with various changes in the brain, including transcription alteration. Here, Bradshaw and colleagues describe the transcriptome of aged human cortical microglia, and show age-related gene expression as related to neurodegeneration.

## Introduction

Recent genetic studies of aging-related neurodegenerative diseases have uncovered many genetic variants that appear to function predominantly through the innate immune system; these results suggest a fundamental role for myeloid cells, including microglia (the resident innate immune cells of the central nervous system) in the pathogenesis of diseases such as Alzheimer’s disease (AD)^1^, Parkinson’s disease (PD)^1^ and amyotrophic lateral sclerosis (ALS)^2^. However, the contribution of microglia to the pathophysiology of these diseases is notoriously difficult to study in mouse, due to the differences in age-related changes in microglial function between mice and humans^3, 4^. The lack of a transcriptome-wide reference profile of human microglia from aged individuals limits our understanding of the aged microglia phenotype and function in humans, particularly in advanced age. Understanding this temporal dimension is critical given that aging is the largest risk factor for late onset neurodegenerative diseases^5^.

In this study, we leverage two prospective cohort studies of aging with brain autopsies^6, 7^, a novel protocol optimized for the isolation of human microglial cells, and cDNA library construction for RNA sequencing (RNA-Seq) from small cell numbers to perform a comprehensive assessment of the transcriptomic landscape of aged human microglia and to create a resource for the community of neurodegenerative disease investigators. We establish the HuMi_Aged gene set that consists of genes primarily expressed in microglia in the aged human brain. This gene set displays many unique functional aspects that are highly relevant to brain aging. Furthermore, we show that this gene set is associated with neuropathological traits and susceptibility genes of AD, confirming the involvement of this cell type in the pathophysiology of the aging brain.

## Results

### Microglia isolation and library construction

We have established an optimized pipeline for the isolation of microglia from post mortem brain samples and an RNA-Seq library construction approach that is specifically suited for use with low cell numbers (described in detail in the Methods section). Using mechanical tissue dissociation followed by magnetic bead based enrichment and fluorescence activated cell sorting (Supplementary Figure 1), this protocol yielded a microglia population, that is free of CD45 high macrophages (Supplementary Figure 2), infiltrating monocytes (Supplementary Figure 2) or other blood cells (Supplementary Figure 3). The resulting RNA-Seq dataset derives from the microglia of ten donors; all microglia were extracted from a sample of dorsolateral prefrontal cortex (DLPFC). All ten subjects had a non-zero burden of amyloid and tau pathology (a characteristic of the aged human brain; Supplementary Figure 4 and Supplementary Table 1), and three of these subjects were diagnosed with AD dementia (Supplementary Table 1).

### Identification of the HuMi_Aged gene set

**Fig. 1:**
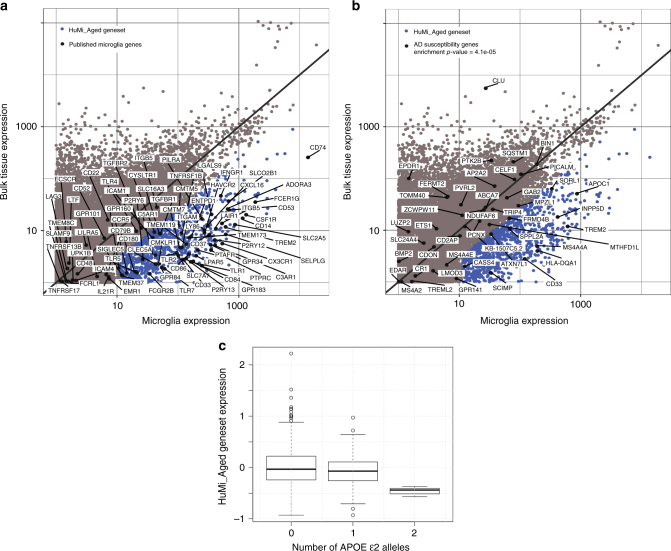
The HuMi_Aged and its relationship to genetic risk of AD. **a** Scatter plot depicting the distribution of gene expression values between the bulk DLPFC (*N* = 540) and the isolated microglia (*N* = 10). Each dot represents a gene. The *X* and the *Y* axes show normalized expression values. One thousand fifty four genes have been identified to be preferentially expressed by microglia (based on the differences in gene expression between the isolated microglia and the bulk cortical tissue, FC > 4) in the aged human brain (dark blue dots). This set of genes has been coined the HuMi_Aged gene set. The HuMi_Aged gene set contained many of the previously established microglia markers in the brain (black dots), such as *CD74*, *CX3CR1*, *P2RY12*, *TREM2* or *GPR34*. **b** Scatter plot showing the distribution of AD risk genes (black dots) in the transcriptomic universe defined by the bulk cortical and the microglial RNA-Sequencing datasets. Each dot represents a gene. The *X* and the *Y* axes show normalized expression values. By using an overrepresentation test, the HuMi_Aged gene set (blue dots) was found to be significantly enriched in Alzheimer’s disease risk genes (black dots; enrichment *p-*value = 4.1e-05). The HuMi_Aged included AD risk genes, such as *CD33*, *TREM2*, *INPP5D*, *APOC1* or *SCIMP*, while other AD risk genes (e.g., *BIN1* or *TREML2*) were found to be not specific to microglia in the aged brain. **c** *APOE* ε2 was associated with reduced expression of HuMi_Aged in the bulk tissue level data (*N* = 540). DLPFC dorsolateral prefrontal cortex, FC fold change

To define a set of genes that are primarily expressed by microglia in the aged human brain, we took advantage of existing DLPFC tissue-level RNA-Seq data^8^ available from an independent set of participants from the same cohorts (*N* = 540). By using a 4-fold increase in microglia:bulk tissue expression as a threshold, we identified 1054 microglia-enriched genes (referred to as “the HuMi_Aged gene set”; Fig. 1a and Supplementary Data 1). Among the microglia-enriched genes we found known markers of microglia (Fig. 1a); many of them in the top 100 expressed genes, confirming the validity of our approach. However, not all previously identified microglia genes were found to be microglia-enriched in our dataset. Interestingly, many genes that were microglia specific in the mouse (e.g., SALL1^9^) were not part of the aged human microglia core transcriptomic signature.

When we compared the HuMi_Aged gene set to other recently published microglia core gene sets^10, 11^ we found that the HuMi_Aged microglia signature had a unique set of genes, not present in other adult^10^ or pediatric^11^ microglia signatures (Supplementary Figure 5a). Among others, pathways associated with DNA damage, telomere maintenance and phagocytosis were significantly enriched in genes that were unique to the aged human microglia signature (Supplementary Figure 5b).

### Enrichment of HuMi_Aged in susceptibility genes

Using series of hypergeometric tests we found that genes in the HuMi_Aged gene set were enriched in susceptibility genes for AD (*p* = 4.1 × 10^−5^) (Fig. 1b) and multiple sclerosis (*p* = 0.0032) but not PD (*p* = 0.82), ALS (*p* = 0.7) or schizophrenia (*p* = 0.83) (Supplementary Figure 6), as defined by the NHGRI-EBI catalog. Nonetheless, a subset of PD (e.g., *SLC50A1*), ALS (*ITPR2*), and schizophrenia (*RENBP*) susceptibility genes were part of the microglia signature in the aged human brain. Interestingly, *RENBP* was not found to be expressed in microglia from middle aged brains^12^. Our findings therefore reinforce the earlier notion, that within the brain, a significant amount of the genetic load that influences the onset of certain neurodegenerative diseases may exert its function through microglia. Focusing on late onset AD (LOAD), we found that many well-known susceptibility genes (*CD33, INPP5D, MS4A4A*, *SORL1, and TREM2)* are part of the HuMi-Aged gene set, while others, such as SPI1^13^, were not. We also identified additional susceptibility genes not known to function in microglia in the context of neurodegeneration (e.g., *SCIMP, SPPL2A*^14^).

### Relationship of the HuMi_Aged to traits and genotypes

We took advantage of the large sample size of the bulk DLPFC RNAseq dataset (*N* = 540) and examined how our HuMi_Aged signature behaved in that tissue-level dataset with respect to traits available in our cohort. For these analyses we created a meta-feature for the HuMi_Aged gene set that resulted in a single value for each participant (Supplementary Data 2). The association of this meta feature was investigated using linear regression. Interestingly, the expression level of the HuMi_Aged gene set in the frontal cortex of the participants increased with advancing age at death (*p* = 0.0012, Table 1), suggesting that the proportion of microglia in the DLPFC may increase with age or that microglia increase the expression of this transcriptional program over time. However, the aged microglia signature is not associated with a diagnosis of AD dementia or a global measure of AD pathology in these tissue-level data; nonetheless, there is a suggestion that it may relate to amyloid deposition (*p* = 0.011). These associations do not change when we perform stratified analyses in subjects with and without a clinical diagnosis of AD (Supplementary Data 3). At the individual gene level, a small number of genes in these ten participants were found to be associated with neuritic plaques (NP), diffuse plaques, and neurofibrillary tangles (NFT) after correction for multiple testing. These genes will require further validation given the modest sample size (*N* = 10) of the present study (Supplementary Figure 7).

**Table 1:** The clinicopathological trait associations of HuMi_Aged

Trait	Estimate	Std. error	*t-*value	*p*-value
Age at death	0.0046	0.0014	3.2633	0.0012
Sex	−0.0399	0.0194	−2.0560	0.0403
Clinical AD	0.0282	0.0193	1.4623	0.1443
Global AD pathology	0.0174	0.0267	0.6500	0.5160
Amyloid load	0.0219	0.0086	2.5575	0.0108
Tau tangle density	0.0021	0.0075	0.2732	0.7848
*APOE* haplotype	0.0061	0.0023	2.6285	0.0088
*APOEe4* count	0.0306	0.0199	1.5412	0.1239
*APOEe2* count	−0.0625	0.0228	−2.7387	0.0064

A meta-feature constructed from the HuMi_Aged gene set revealed its association with age at death and *APOE* ε2 in the bulk tissue RNA-Seq dataset (*N* = 540). Please note, the reference sex was male—women have a greater HuMi_Aged signature, on average. Five hypotheses were tested in primary analysis, accordingly the significance threshold was *p* < 0.01

From a genetic point of view, the *APOE* haplotype is associated with the HuMi_Aged meta-feature (*p* = 0.0088), and, interestingly, the effect appears to be driven not by *APOE* ε4 but by *APOE* ε2, the haplotype that is protective for AD. In our tissue-level data, *APOE* ε2 was associated with reduced expression of HuMi_Aged (Fig. 1c) (*p* = 0.0064), consistent with the predicted direction of effect.

### Identification of human microglia genes affected by aging

**Fig. 2:**
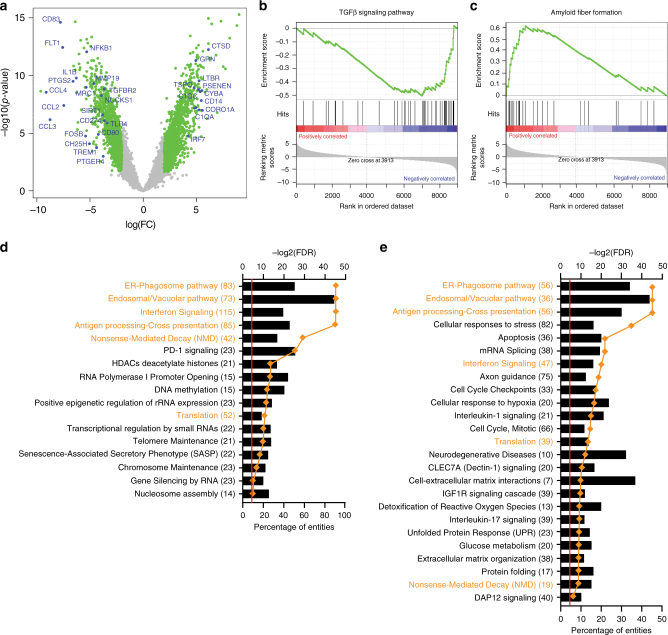
The phenotype of aged human microglia. Functional annotation reveals that human microglia aging manifests itself as loss of function as well as gain of function changes in phenotype. **a** Volcano plot depicting the results of differential gene expression analysis between the Zhang et al. (middle aged microglia; *N* = 3) and our dataset (aged microglia; *N* = 10). One thousand sixty genes were found to be upregulated while 1174 genes were downregulated with aging. **b**, **c** GSEA enrichment plots of representative gene sets that were significantly enriched in either downregulated (**b**) or upregulated (**c**) genes. The TGFβ signaling pathway gene set was enriched in downregulated genes (loss of function with aging, (**b**)) while the Amyloid fiber formation pathway was enriched in upregulated genes (gain of function with aging, (**c**)). **d** Pathway analysis of the genes that were significantly upregulated with aging in microglia in the two RNA-Seq datasets. **e** Pathway analysis of the proteins detected with a shotgun proteomic approach in aged human microglia. Please note the significant overlap between the functional phenotypes revealed by the two approaches (overlapping REACTOME entities highlighted in orange). Legends in **d** and **e**: orange diamonds (–log2(FDR)), columns (Percentage of entities). GSEA gene set enrichment analysis, FDR false discovery rate

In order to identify the microglia genes that are affected by aging in the human cortex, we took advantage of another published adult human microglia transcriptomic dataset, that is derived from middle aged individuals^12^. We performed an exploratory study comparing the transcriptomic profiles of middle aged (mean age = 53, SD = ±5.29, *N* = 3) and our aged (mean age = 94.07, SD = ±0.95, *N* = 10) human microglia (Supplementary Figure 8a). By assessing only those genes that were reliably detected in both datasets (Supplementary Figure 8b; Materials and Methods), we identified 1060 genes that were up-regulated (logFC > 2, adjusted *p-*value > 0.05), and 1174 genes that were down-regulated in our aged human microglia (Fig. 2a, Supplementary Data 4). Importantly, both datasets were highly enriched in microglia genes (Supplementary Figure 8c). Intriguingly, gene set enrichment analysis (GSEA) of the differentially expressed genes identified the Amyloid fiber formation (Fig. 2b) REACTOME gene set to be enriched for genes that are up-regulated in aged human microglia, while the TGFβ signaling KEGG pathway (Fig. 2c) was enriched for down-regulated genes. These results suggest that human microglial aging may result both in gain of function as well as in loss of function of certain transcriptional programs; diminished TGFβ signaling highlights the perturbation of homeostatic programs as microglia activate reactive pathways to respond to aging-related changes such as the accumulation of amyloid pathology.

### The proteome of aged human microglia

To assess whether our transcriptomic approach faithfully captured genes that were expressed at the protein level in microglia and could therefore affect cellular function, we also generated a proteomic profile from one sample of 5000 purified microglia isolated with the same pipeline that was used to produce the RNAseq dataset. Using LC-MS mass spectrometry, we generated a shotgun proteomic profile consisting of 640 proteins for which we have a measure of relative expression. As expected, the detected proteins were distributed along the gene expression spectrum, with a skew towards the more abundant gene transcripts (Supplementary Figure 9a). By comparing the expression levels of proteins and mRNA, we found that these two measures were considerably correlated (*r*_S_ = 0.3112, *p* < 0.0001) (Supplementary Figure 9b). Twenty seven of the detected proteins were also part of HuMi_Aged gene set. Gene ontology analysis of the detected proteins revealed some of the same REACTOME pathways that we found to be up-regulated in the aged human microglia transcriptome, such as the ER-Phagosome pathway (FDR = 3.19E-14) (Fig. 2d, e). The list of proteins detected in aged human microglia is available in Supplementary Data 5.

## Discussion

Here, we present a novel microglia isolation protocol and RNA-Seq library construction approach to create transcriptomic profiles from autopsy-derived microglia using only 5000 cells per individual. This number has been previously empirically established in our laboratory to be the lowest cell number at which the stochastic drift in population mean is no longer substantial. Given that current transcriptomic approaches require hundreds of thousands of microglia cells^10, 11^ and the availability of fresh autopsy samples from well-characterized subjects is very limited, the protocol presented here will be a useful tool for the scientific community in order to most efficiently utilize the available precious human brain specimens, especially in the case of samples where viability is compromised, such as those affected by chronic neurodegeneration and/or aging. Furthermore, we provide the scientific community with the first transcriptomic atlas of aged human microglia (publicly available at http://shiny.maths.usyd.edu.au/Ellis/MicrogliaPlots website). This RNA-Sequencing dataset is derived from quasi-centenarian donors of the ROS and MAP cohorts.

It was beyond the scope of this study to discriminate between microglia-derived macrophages and infiltrating monocyte-derived macrophages, primarily due to the current lack of markers that would reliably allow us to make that distinction in human samples. Nonetheless, the lack of a CD45high subpopulation in our preparations further supports that our microglia preparations are largely devoid of blood-borne monocytes and perivascular macrophages, both of which would have shown up on the scatter plots as a small CD11b+/CD45high subpopulation on top of the CD11b+/CD45+ microglia population. Furthermore, the high expression of the microglia specific genes (many of whose expression was close to the 100th percentile) confirms that blood borne monocyte contamination was negligible; the lack of considerable CCR2 expression in our dataset shows that our preparations were also free from recently recruited tissue infiltrating inflammatory monocytes.

By combining our microglia transcriptomic profiles with existing cortical RNAseq data from an independent set of subjects from the same cohort, we derived the HuMi_Aged gene set and have illustrated the utility of this resource by showing that it captures an important fraction of genetic susceptibility to both AD and MS. In particular, we report the intriguing result that the *APOEε2* haplotype is associated with a reduced HuMi_Aged signature, consistent with its role as a protective factor in AD, and this observation aligns with recent reports that *APOE* may interact with microglial genes such as *TREM2*^15–17^. *APOEε4* is a much stronger risk factor for AD but does not appear to be related to this microglial signature and most likely exerts its effect through another mechanism.

Importantly, we also found that the HuMi_Aged core microglia profile displayed a number of differences in relation to the already available pediatric and adult microglia signatures. Functional annotation of the genes that were unique to the aged human microglia signature presented here revealed that cellular and organismal aging related pathways and biological processes were primarily affected. These findings confirm the notion, that the aged human brain harbors a distinctive microglia phenotype, whose identity is shaped by cell autonomous/intrinsic (DNA damage response, telomere maintenance) as well as extrinsic (decline in homeostatic and increase in phagocytic signaling from the brain parenchyma) factors that are unique to the aging brain environment.

Thus, the HuMi_Aged gene set can be used to more accurately explore the role of microglia in other tissue-level datasets from older brains. Increasing our sample size will allow us to more directly dissect the contribution of microglia to brain aging and neurodegenerative diseases as current tissue level profiles probably under-estimate the contribution of microglia which makes up a small proportion of the tissue and often have relatively small amounts of RNA. Nonetheless, this resource will be a critical reference for efforts to optimize in vitro systems (using either monocytes^18^ or induced pluripotent stem cell-derived microglial like cells^19–21^) for neurodegenerative disease studies. Finally, our initial proteomic profile not only validates our transcriptomic data but also presents a number of proteins that could be targets for developing novel markers of human microglia in histological studies and for the development of new PET ligands with which to assess the immunologic state of the aged human brain.

## Methods

### Cohorts and donors

The biological specimens used in this study originated from two prospective studies of aging: the Religious Orders Study (ROS)^6^ and the Memory and Aging Project (MAP)^7^. Participants have to be at least 53 (ROS) or 55 (MAP) years old and non-demented at enrollment and sign an Anatomical Gift Act agreeing to donate their brain and spinal cord at the time of death. Thus, they represent a sample of the older population from the Chicago, IL metropolitan area. Each subject undergoes annual neuropsychologic evaluations while alive and a structured, quantitative neuropathologic examination at autopsy. All brain autopsies, experiments, and data analysis were done in compliance with protocols approved by the Partners Human Research Committee and the Rush University Institutional Review Board. The data presented in this study originates from ten donors. The clinicopathological characteristics of the participants are shown in Supplementary Table 1. Their mean age at death was 95 years and 80% of them were female. Nine out of ten of the participants showed a range of amyloid and tau pathology accumulation that is typical in this type of older, population-based sample. Three of the donors were ante mortem diagnosed with clinical AD.

### Brain autopsy and shipping of brain specimens

To obtain the midfrontal gyrus (BA 9/46) a 1.5 cm × 1.5 cm thick section is cut off from the first cerebral slab anterior to the caudate. After weighing, the tissue is placed in ice-cold transportation medium (Hibernate-A medium (Gibco, A1247501) containing 1% B27 serum-free supplement (Gibco, 17504044) and 1% GlutaMax (Gibco, 35050061)) and shipped overnight at 4 °C with priority shipping. Brain specimens were distributed for this project from autopsies taking place Sunday morning to Thursday. Only autopsies for which the post mortem delay was less than 12 h were included in this study.

### Microglia isolation and sorting

Upon arrival of the autopsy brain sample, the cerebral cortex and the underlying white matter were dissected under a stereomicroscope. All procedures were performed on ice. Only microglia isolated from the grey matter were used in this study. The dissected tissue was placed in HBSS (Lonza, 10-508F) and weighed. Subsequently the tissue was homogenized in a 15 ml glass tissue grinder—0.5 g at a time. The resulting homogenate was filtered through a 70 μm filter and spun down with 300 g for 10 min. The pellet was resuspended in 2 ml staining buffer (PBS (Lonza, 17-516F) containing 1% FBS) per 0.5 g of initial tissue and incubated with anti-myelin magnetic beads (Miltenyi, 130-096-733) for 15 min according to the manufacturers specification. The homogenate was than washed once with staining buffer and the myelin was depleted using Miltenyi large separation columns. The cell suspension was spun down and incubated with anti-CD11b magnetic beads (Miltenyi, 130-049-601, clone M1/70) for 15 min. Following a wash with staining buffer the CD11b+ cells were isolated on a Miltenyi MS column while the CD11b− fraction was cryopreserved using FBS containing 10% DMSO. The CD11b positive fraction was than incubated with anti-CD11b AlexaFluor488 (BioLegend, 301318, clone ICRF44) and anti-CD45 AlexaFluor647 (BioLegend, 304018, clone HI30) antibodies as well as 7AAD (BD Pharmingen, 559925) for 20 min on ice. Subsequently the cell suspension is washed twice with staining buffer, filtered through a 70 μm filter and the CD11b+/CD45+/7AAD− cells (Supplementary Figure 2b) were sorted on a BD FACS Aria II sorter. Cells were sorted in A1 well of a 96 well PCR plate (Eppendorf, 951020401) containing 25 μl of TCL buffer (Qiagen, 1031576) with 1% beta mercaptoethanol (Sigma, M3148). Following FACS the lysate was vigorously vortexed for 30 s, spun down, snap frozen on dry ice and stored at −80 °C until further processing.

### Library construction and sequencing

Library construction was performed using the SmartSeq-2 protocol described in detail elsewhere^22^ with minor modifications. Briefly, the 96 well plates (Eppendorf, 951020401) were thawed on ice and spun down. *RNA Isolation*. 2.2 × volume of RNA-SPRI (Beckman Coulter, A63987) beads were added to the cell lysate, mixed, incubated for 10 min at room temperature and placed on a magnet (Thermo Fischer, 12027) for 5 min. The supernatant was removed and the beads were washed with 80% ethanol (VWR, 89125-170) three times, let to dry on air for 10 min on the magnet and subsequently resuspended in Master Mix 1 (containing RT primers 10 μM (IDT—Custom, sequence below), dNTP mix 10 mM (Thermo-Fischer, R0192) and RNase-inhibitor 4 U/μl (Clontech, 2313B) in water). The plate is then incubated at 72 °C for 3 min in a thermocycler and then placed on ice. *Whole Transcriptome Amplification*. Subsequently the beads are dispersed in Master Mix 2 (containing Maxima Buffer (Thermo-Fischer, EP0753), betaine 5M (Sigma-Aldrich, B0300-1VL), MgCl_2_ 10 mM (Sigma-Aldrich, M1028), TSO 10 μM (Exiqon Custom, sequence below), RNase inhibitor 40 U/μl, Maxima RNaseH-minus RT 200 U/μl in water) and a PCR run is performed on the thermocycle with the following parameters: Hold (42 °C, 90 min), 10 cycles (50 °C, 2 min; 42 °C, 2 min), Hold (70 °C, 15 min). *PCR Preamplification*. The plate is then removed from the thermocycle, Master Mix 3 (containing ISPCR primer 10 μM (IDT custom, sequence below) KAPA HiFi HotStart ReadyMix (KAPA BioSystems, KK2602) in water) is added, and run again with the following parameters: Hold (98 °C, 3 min), 14 cycles (98 °C, 15 s; 67 °C, 20 s; 72 °C, 6 min), Hold (72 °C, 5 min). *DNA Cleanup*. The PCR products were purified with 0.8 × AMPure XP SPRI beads (Beckman Coulter, A63880). The AMPure XP SPRI beads were added to the sample, mixed thoroughly, incubated at room temperature for 6 min and placed on the magnet for 6 min. The supernatant was pipetted off and the beads were washed with 70% ethanol 2 times and than let to dry on room temperature for 10 min. The material was eluted from the beads in TE (TEKNOVA, T0228), removed the beads from the eluent with a magnet and the eluent was than transferred to a new 96 well PCR plate for a second round of PCR product purification with AMPure XP SPRI beads. *Quality control*. The quality and the quantity of the cDNA were assessed with Qubit DNA HS (Life Technologies, Q32854) and BioAnalyzer DNA HS assays (Agilent, 5067-4626). *Library construction*. All samples were diluted to the concentration range of 0.1–0.2 ng/μl in order to be used with the Nextera XT kit (Illumina, FC-131-1096) for library construction. In a fresh plate TD, ATM and the input sample were mixed and than placed in a thermocycler for the following program: Hold (55 °C, 10 min), Hold (10 °C, ∞); following which it is placed on ice. Subsequently NT, NPM, and mixed indices were added to the samples and the plate was run using the following parameters: Hold (72 °C, 3 min), Hold (95 °C, 30 s), 12 cycles: (95 °C, 10 s; 55 °C, 30 s; 72 °C, 60 s), Hold (72 °C, 5 min), Hold (4 °C, ∞). *Sample cleanup*. Subsequently the samples were pooled and DNA cleanup was performed by adding 0.9 × volume of AMPure XP SPRI beads, incubating for 5 min on room temperature, placing the sample on a magnet for 5 min, washing the beads twice with 80% ethanol and then eluting the cDNA with TE. The cleanup step is repeated once. *Quality control*. The quality and the quantity of the cDNA were assessed with Qubit DNA HS and BioAnalyzer DNA HS assays. *Sequencing*. The pool of ten samples was sequenced on one lane of an Illumina HiSeq2500 flowcell with a read length of 100 bp and paired end reads.

Primers used:

RT Primer−5′−AAGCAGTGGTATCAACGCAGAGTACT30VN−3′

TSO−AAGCAGTGGTATCAACGCAGAGTACATrGrG+G

ISPCR−5′−AAGCAGTGGTATCAACGCAGAGT−3′

### Quality control and data processing of RNA-Seq data

RNA-Seq reads in FASTQ format were inspected using FASTQC program. Barcode and adapter contamination, low quality regions (10 bp at beginning of each fastq reads) were trimmed using FASTX-toolkit. We then used Tophat (2.1.0) to align trimmed reads to human reference genome sequences (hg19) and transcript annotation from Gencode (v19) then applied Cufflinks (2.2.1) to estimate gene and isoform abundance. The estimation of abundance is in FPKM format. We then performed the following normalization approach: first, we filtered out low expressed genes with FPKM < 0.1 in more than two samples, resulting in expression levels for 9489 genes. Then we used voom method in limma^23^ package to normalize filtered FPKM values.

### Identification of microglia enriched genes

Given that microglia constitute only 12% of the cells in the prefrontal cortex and are known to have very low RNA content, our argument was that genes that will be significantly highly expressed in the isolated microglia samples, when compared to the bulk, will be most likely primarily expressed by microglia. To make the microglia and bulk samples more comparable the mapped genes were first filtered to only include those with a uniprot identifier. Further normalization was then performed, by comparing the log gene expression of a sample to the average of all samples, a robust linear model was used to normalize out any residual GC content and gene length artifacts. Then, by contrasting the bulk RNA-Seq data with the gene expression profiles of isolated microglia and using a fold change cut off of 4, we identified 1054 genes that were enriched in microglia (Fig. 1a; FC genes in Supplementary Data 1). Seven hundred seventy one genes reached statistical significance (PVAL genes; Supplementary Data 1). Statistical significance depends on the variability between samples, and in our case, this might originate from the microglial responses to pathological features present in the brains of the donors, that had varying amounts of amyloid load and tau pathology (Supplementary Table 1). In order not to exclude the genes that are responsive to the aging brain environment, we decided to use the FC genes for downstream analysis.

### Over-representation analysis

The enrichment of the HuMi_Aged gene set in genes associated with neurodegenerative disease and psychiatric disorders was tested with an over-representation analysis. Genes that had previously been associated with neurodegenerative disease and psychiatric disorders were sourced from the NHGRI-EBI Catalog of published genome-wide association studies^24^.

### Association with traits

A meta-feature was created to capture the change in expression of the HuMi_Aged gene set as a collective in the bulk tissue and assess its association with various AD an aging associated traits. To do this each gene was standardized to have variance equal to 1 and then the expression of all genes in the HuMi_Aged gene set was averaged over each sample. A series of *t*-tests were then used to test for association of the meta-feature with various clinical, pathological and mutation variables.

### Differential gene expression

We contrasted the gene expression levels of aged human microglia with a previously published microglia transcriptomic dataset derived from middle-aged individuals (NCBI GEO accession number: GSE73721)^12^. The differential gene expression analysis was performed using limma package^23^. We identified 2234 genes (logFC > 2 and logFC<−2, adjusted *p*-value < 0.05, Supplementary Data 4) that were differentially expressed in aged human microglia.

### GSEA and pathway analysis

GSEA was performed by pre-ranked GSEA module in GenePattern^25^. Pathway analysis was performed using the publically available platform REACTOME with default setting (www.reactome.org).

### Proteomic analysis

For proteomic analysis microglia were isolated as described above. 5000 CD11b+/CD45+/7AAD- cells were sorted in A1 well of a 96 well plate containing 100 μl of PBS. Following FACS the cells were spun down after which all but 10 μl of PBS was removed from A1 well and the plate was than snap frozen on dry ice and stored at −80 °C until further processing. Sorted cell pellets were lysed and extracted by adding 10 μL of homogenization buffer (8 M urea, 10 mM dithiothreitol, 50 mM Tris pH = 8) followed by 30 s in a bath sonicator. The lysate was then incubated at 37 °C for 30 min to denature and reduce the proteins. Protein solution was quantitatively transferred to a low retention LC vial (Waters) using 20 μL of 50 mM Tris pH = 8. SNaPP analysis was carried out as described previously^26, 27^. Briefly, 25 μL of sample was injected onto a 150 µm × 2 cm immobilized enzyme reactor for digestion. Following digestion, peptides were separated using a 180 min gradient on an in-house 50 µm × 75 cm C18 (Phenomenex) analytical column. The SNaPP system was coupled to an Orbitrap Fusion Lumos mass spectrometer (Thermo Scientific) operated in top speed mode. Fragmentation was carried out in the ion trap with maximum injection time of 250 ms for MS2 to increase sensitivity for low intensity ions. The label-free data was quantified using the iBAQ (intensity based absolute quantification) approach^28^.

### Immunohistochemistry

Immunohistochemistry was performed as described elsewhere^29^. Briefly brains were removed and processed according to a standard protocol. One hemisphere was cut coronally into 1 cm slabs and fixed in 4% paraformaldehyde. β-amyloid deposits and PHFtau tangles were assessed in 20 µm sections. Immunohistochemistry and computer-assisted image analysis were used for β-amyloid (clone 4G8, 1:9000 Covance Labs, Madison, WI) and stereology was used for PHFtau tangles (clone AT8, 1:2000; ThermoScientific, Waltham, MA) across eight different brain regions including the entorhinal cortex, the hippocampus at CA1, superior frontal cortex (Broadmann area [BA] 6/8), mid frontal cortex (BA 46/9), inferior temporal cortex (BA 20), angular gyrus cortex (BA 39/40), cingulate gyrus (BA 32/33) and calcarine cortex (BA 17). β-amyloid load and the density of PHFtau tangle density was obtained by averaging the mean percentage area per region, across all regions. The absence of β-amyloid was characterized by a β-amyloid load of zero in all the eight immunostained sections. NP and NFT were identified with modified Bielshowsky stain in 6 µm sections in five different regions (entorhinal cortex, hippocampus at CA1, midfrontal cortex, middle temporal gyrus, and inferior parietal cortex). CERAD criteria assessed NP burden and a Braak staging assessed the distribution and severity of NFT pathology. The diagnosis of pathologic AD was based on the National Institute on Aging (NIA)–Reagan criteria (1997).

### Technical considerations

Since the brain tissue is obtained at autopsy with a postmortem delay of, on average, 7 h, the release of surviving monocytes from the clotted blood present in the blood vessels of the brain, with the non-enzymatic approach used in this study, is exceedingly unlikely. The high expression of the markers previously reported to be microglia specific when compared to monocytes (e.g., *P2RY12*, *TMEM119*, *TREM2*, *GPR34*, *CX3CR1*)^30–32^ in our preparations (Fig. 1a and Supplementary Figure 8c) further supports this scenario. The lack of CD11b−/CD45+ cells on the flow cytometry scatter plots (Supplementary Figure 2b) further supports the idea, that there was no blood borne immune cell contamination in our preparations. Nonetheless, it was beyond the scope of this study to discriminate between microglia derived macrophages and infiltrating monocyte derived macrophages, primarily due to the current lack of technologies that would reliably allow us to make that distinction in human samples. Despite the differences in the mode of isolation (CD11b/CD45 based FACS versus CD45 based immunopanning), there was an almost perfect overlap between the Zhang and our dataset in terms of the expression levels of previously established microglia marker genes, since in both datasets they scored close to the 100th percentile (Supplementary Figure 8c). This increases our confidence that the observed differences in gene expression are, with some contribution from potential regional and disease specific differences, primarily due to organismal aging and/or cellular senescence.

### Data availability

The RNA-Sequencing data originating from the bulk dorsolateral prefrontal cortex (DLPFC; 540 donors) and from purified microglia (10 donors) from the same brain region is available at Synapse (https://www.synapse.org/#!Synapse:syn3219045 and syn11468526). The microglia RNA-Sequencing dataset is also available and searchable at http://shiny.maths.usyd.edu.au/Ellis/MicrogliaPlots/. Please note, that the searchable platform will ultimately move to the website of the Center for Translational and Computational Neuroimmunology (at Columbia University Medical Center, New York), which is currently under development. For all other inquires please contact the corresponding authors.

## Electronic supplementary material

Supplementary Information

Description of Additional Supplementary Files

Supplementary Data 1

Supplementary Data 2

Supplementary Data 3

Supplementary Data 4

Supplementary Data 5
