# Supplementary material for: A transcriptomic atlas of aged human microglia
Source: Nat Commun. 2018 Feb 7;9:539. doi: 10.1038/s41467-018-02926-5 (PMC5803269; doi:10.1038/s41467-018-02926-5)
Supplement: Supplementary file 1 — Supplementary Information [file 41467_2018_2926_MOESM1_ESM.pdf]

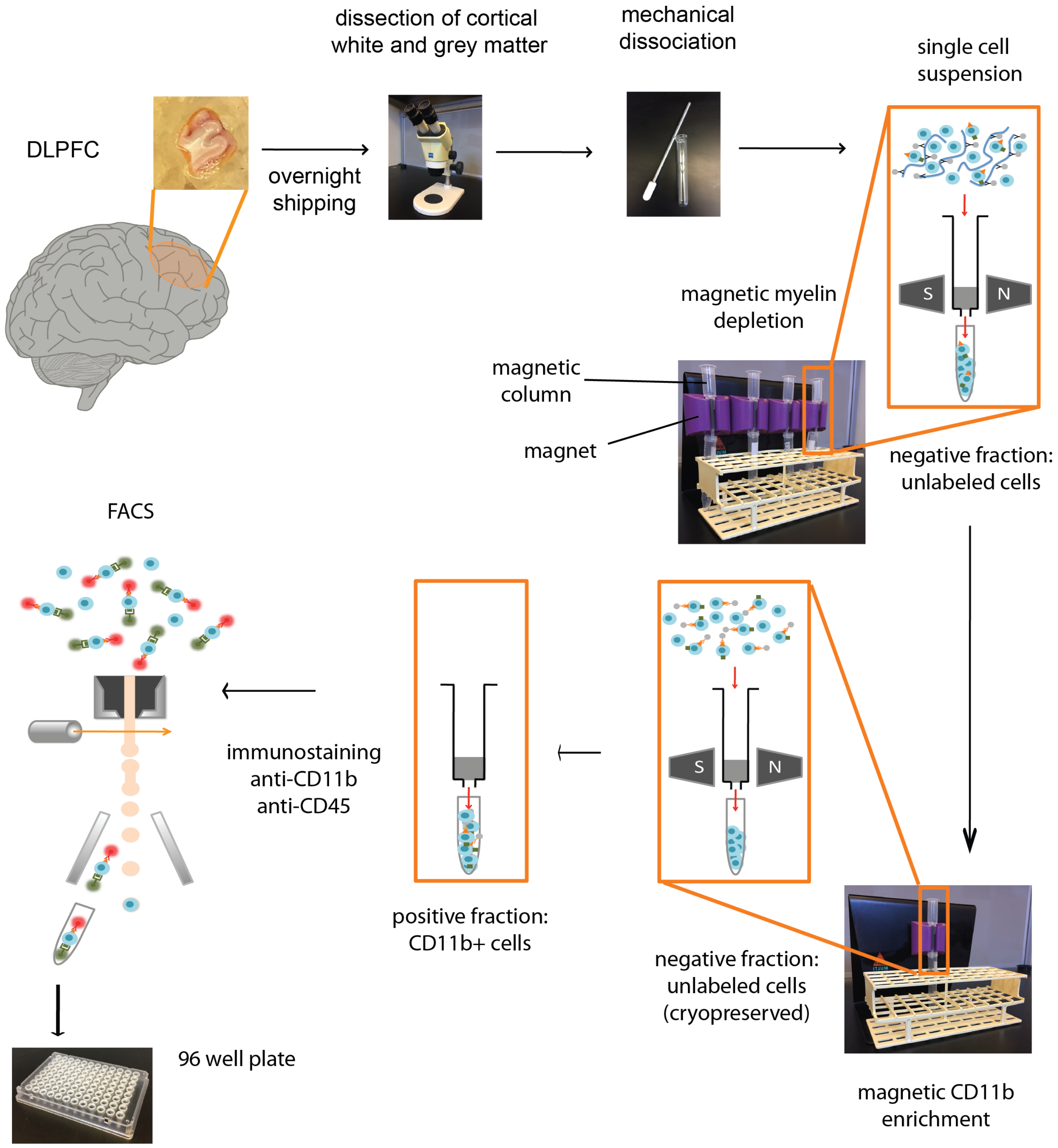


**Supplementary Figure 1. Workflow of microglia isolation.** A 1.5 cm x 1.5 cm piece of the DLPFC (BA9/46) was shipped to us in transportation medium (Hibernate A containing 1% B27 serum free supplement and 1% GlutaMax). The cerebral cortex was separated from the underlying white matter under a stereomicroscope. The tissue was mechanically dissociated with a tissue grinder, the myelin was depleted with anti-myelin magnetic beads and the resulting cell suspension was subjected to microglia enrichment using positive selection with anti-CD11b magnetic beads. The microglia cells were than further purified with FACS. Abbreviations: DLPFC dorsolateral prefrontal cortex; FACS fluorescence activated cell sorting.

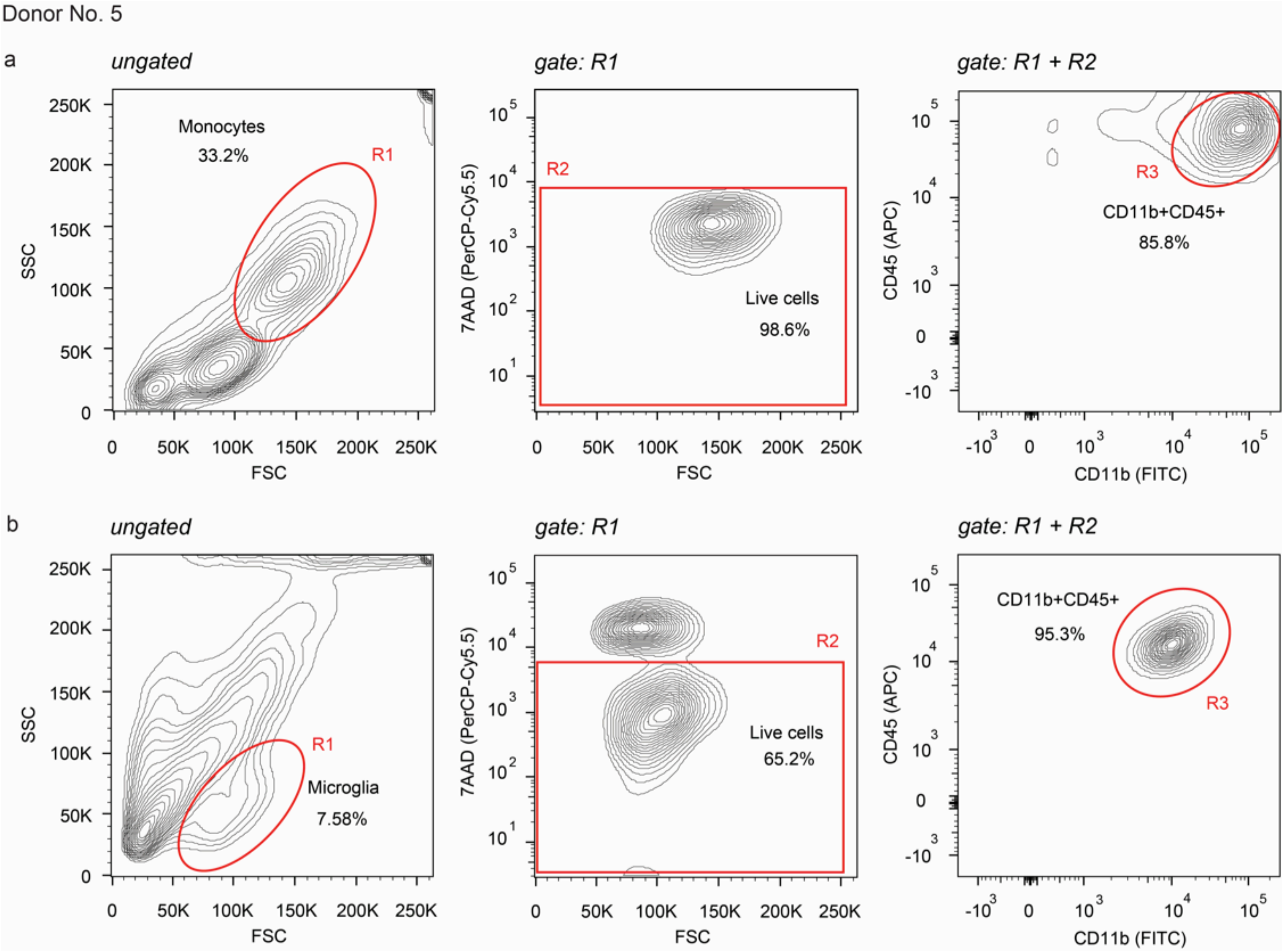


**Supplementary Figure 2. Quality control and gating strategy.** (a) Contour plots depicting a PBMC sample stained, analyzed and sorted in the same way as the microglia preparations. Please note the higher expression of CD11b and CD45 on monocytes and other immune cells, when compared to microglia in (b). (b) Contour plots showing the FACS gating strategy for the microglia purifications. Microglia were purified by FACS from a cell suspension enriched in CD11b+ cells. The first gate on the FSC/SSC roughly corresponds to the traditional lymphocyte gate (small cells). Microglia were sorted based on their characteristic CD11b high and CD45 intermediate expression profile. 5000 CD11b+/CD45+/7AAD- microglia were sorted into A1 of a 96 well plate in a buffer depending on downstream application. Please note the absence of any CD11b-/CD45+/7AAD- cells, upholding the notion that there were no blood derived cells in our preparations. Please note the lack of a CD45high macrophage subpopulation within our preparation. 7AAD is a DNA intercalator that is excluded from live cells. Only microglia isolated from the cerebral cortical part (grey matter) was used in this study. The PBMC sample used in (a) and the microglia sample used in (b) were from the same donor (Donor No. 5 – see Supplementary table 1). Abbreviations: DLPFC dorsolateral prefrontal cortex; FACS fluorescence activated cell sorting; FSC forwards scatter; SSC side scatter; 7AAD 7-aminoactinomycine; PerCP-Cy5.5 a fluorochrome, denoting a fluorescent channel here; APC a fluorochrome, denoting a fluorescent channel here; FITC a fluorochrome, denoting a fluorescent channel here.

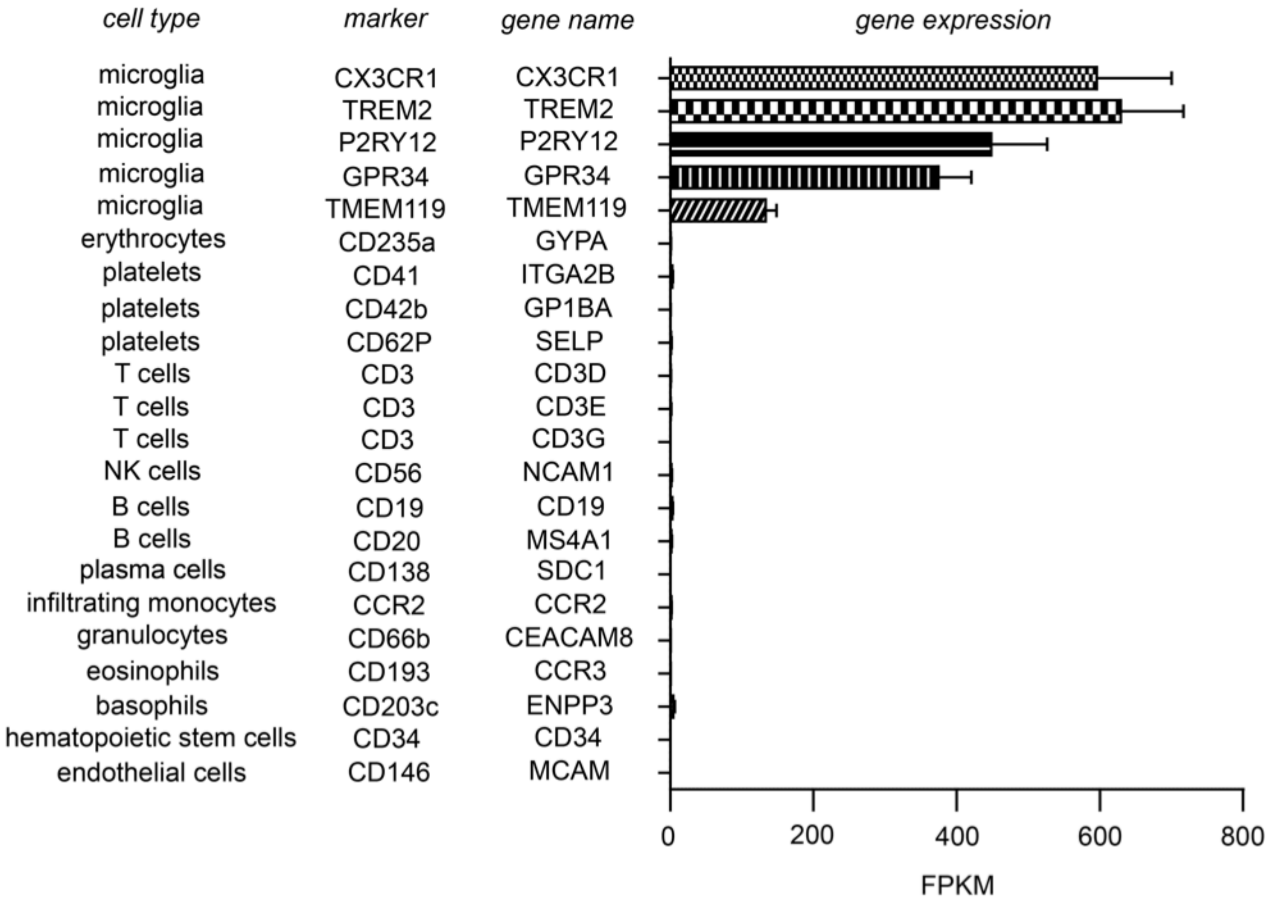


**Supplementary Figure 3. Lack of contaminating blood borne cells.** The preparations were highly enriched in microglia, without contamination from blood borne cells. The bar graphs depict the expression values of the listed markers in the aged human microglia RNA-Sequencing dataset (N=10). Please note the high expression levels of the microglia markers and the lack of expression of markers for other immune cell types or blood borne cells. The lack of CCR2 expression also suggests, that recently infiltrated inflammatory monocyte were not a substantial source of contamination in our preparations. Error bars represent the SEM. Abbreviations: FPKM Fragments Per Kilobase of transcript per Million mapped reads.

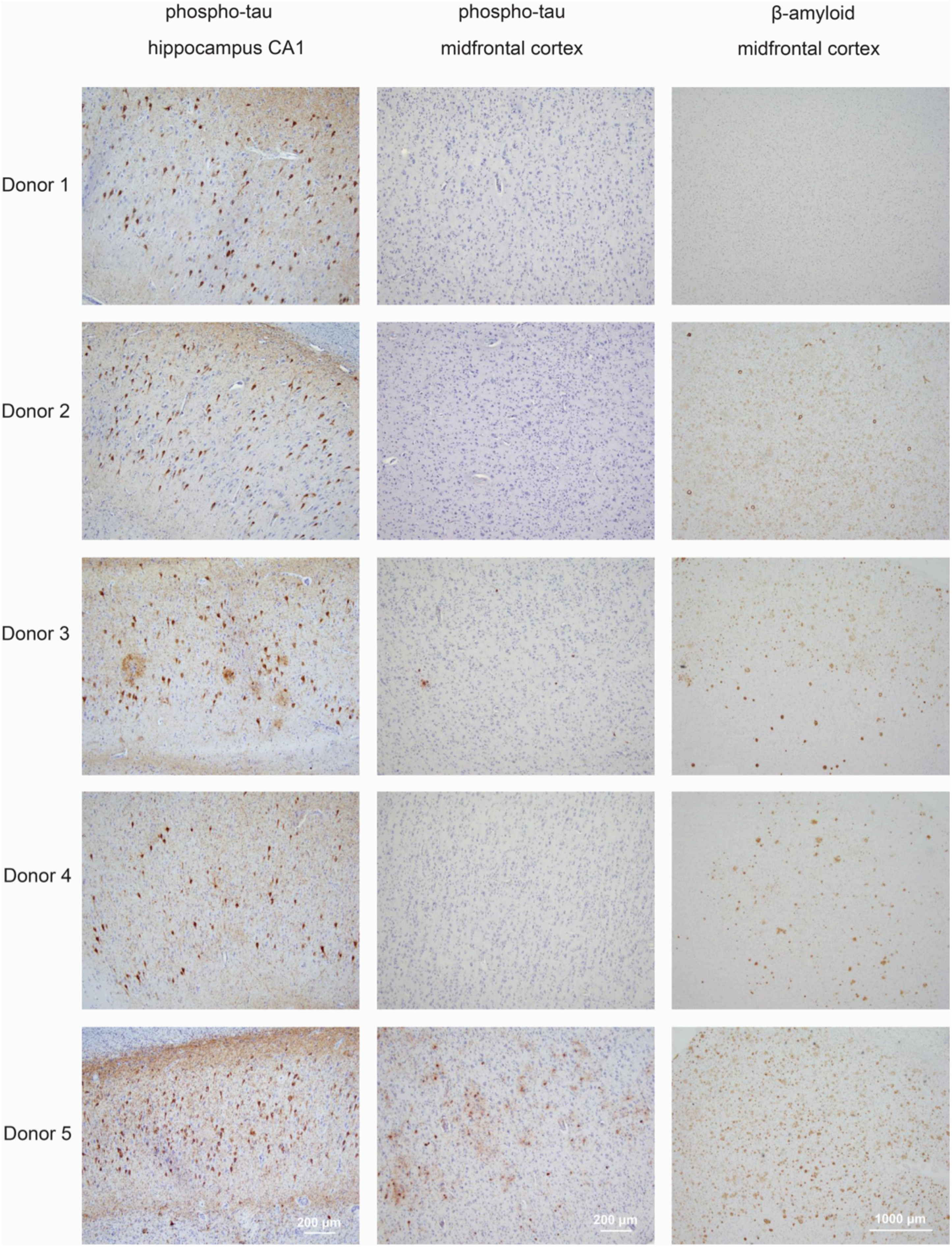

phospho-tau
hippocampus CA1
phospho-tau
midfrontal cortex
β-amyloid
midfrontal cortex
Donor 1
Donor 2
Donor 3
Donor 4
Donor 5
200 µm
200 µm
1000 µm

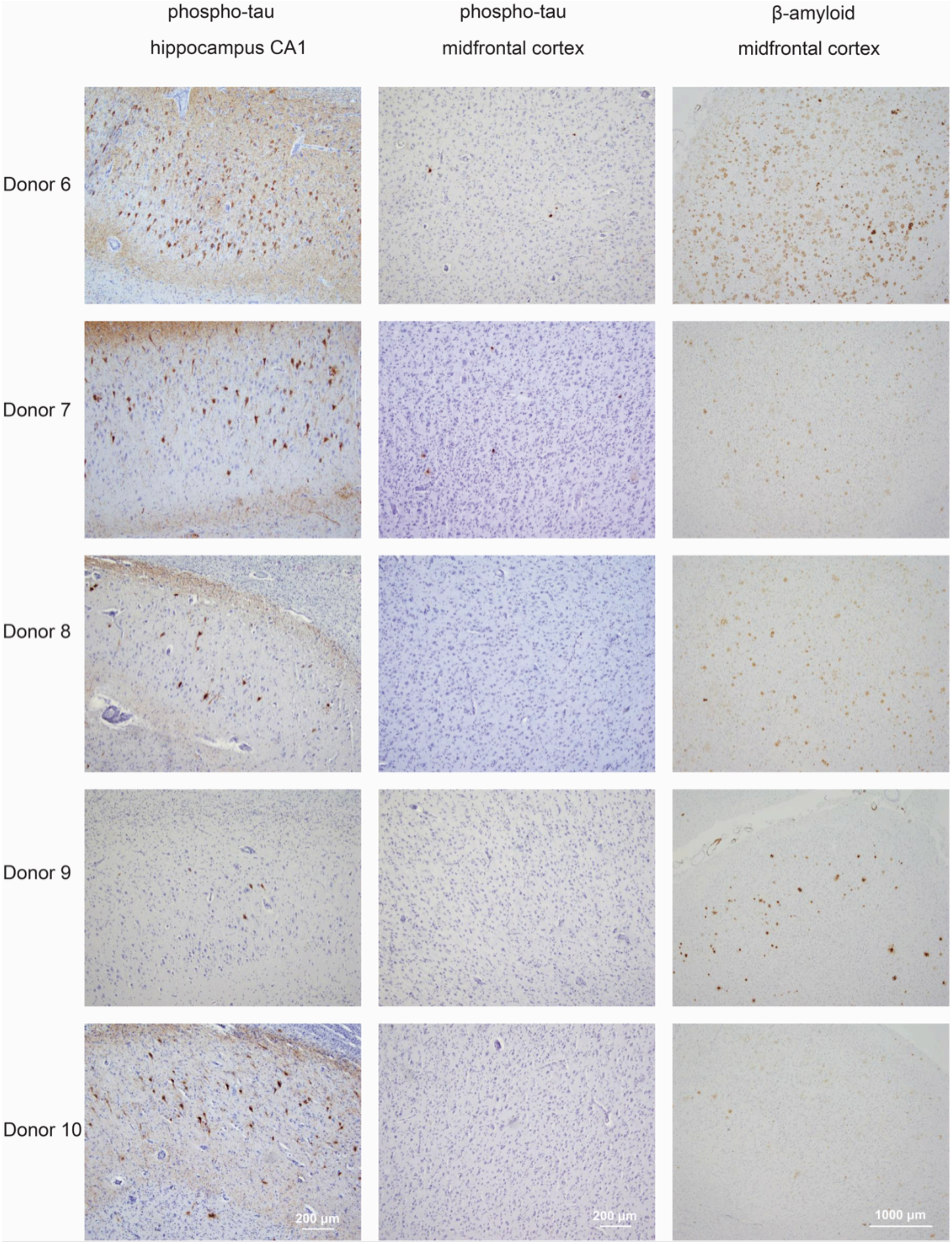

phospho-tau
hippocampus CA1
phospho-tau
midfrontal cortex
β-amyloid
midfrontal cortex
Donor 6
Donor 7
Donor 8
Donor 9
Donor 10
200 μm
200 μm
1000 μm

**Supplementary Figure 4. Immunohistochemical analysis of the pathological burden.** Two brain regions were analyzed from the brains of the donors included in this study: CA1 region of the hippocampus and midfrontal cortex. The monoclonal antibody clone AT8 was used to detect hyperphosphorylated tau protein, while β-amyloid deposits were detected with monoclonal antibody clone 4G8. Please note, that none of the donors had a zero burden of tau pathology in the CA1 region and a zero burden of β μ-amyloid deposition in the midfrontal cortex. Scale bars represent 200 μm for the tau micrographs, and 1000 μm for the beta-amyloid micrographs. (a) Donors 1-5. (b) Donors 6-10.

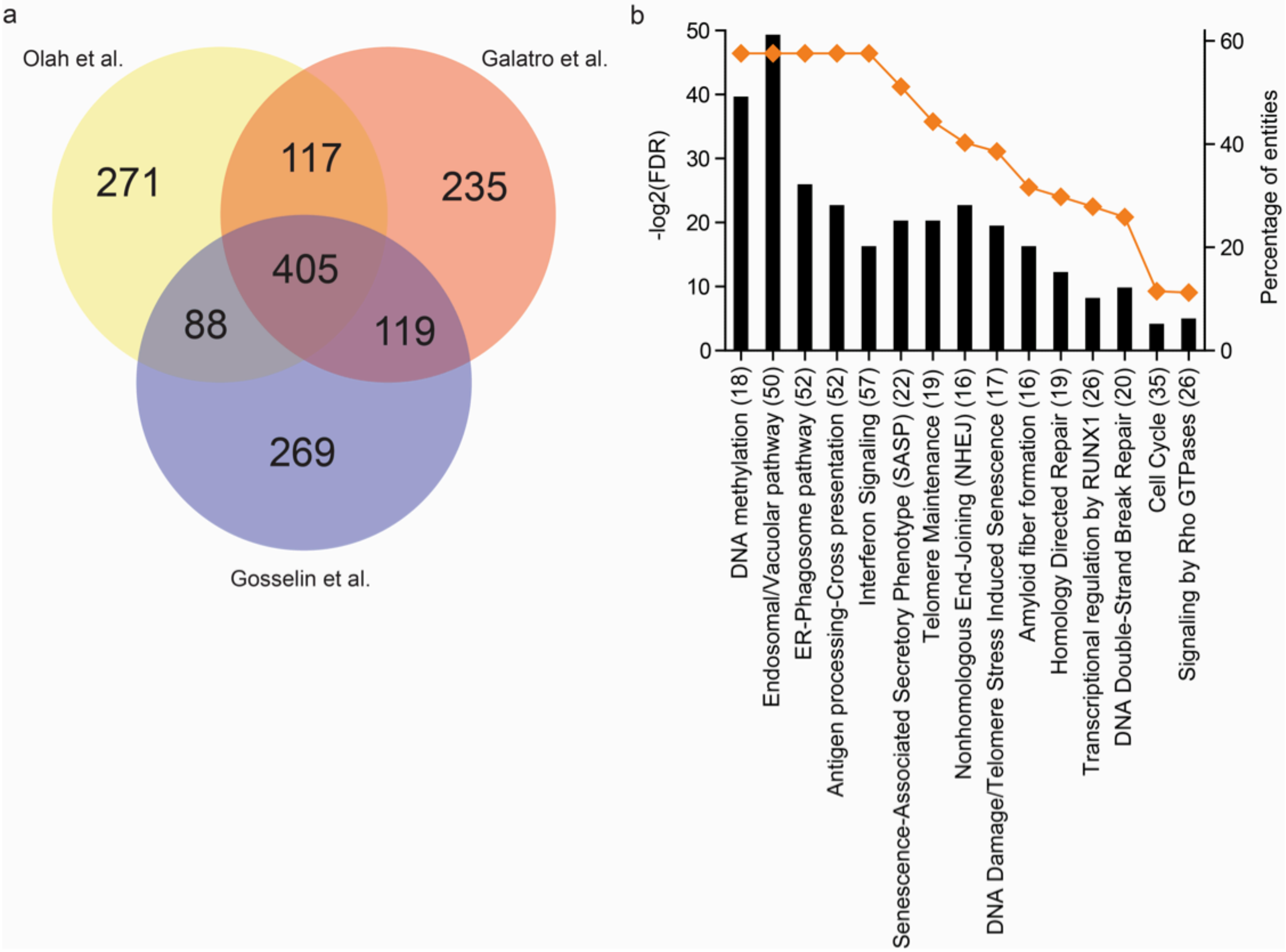


**Supplementary Figure 5. Comparison of HuMi_Aged with other microglia signatures.** (a) Venn diagram depicting the overlap between the three recently established microglia core signatures: a pediatric microglia signature (Gosselin et al.), a core microglia signature from primarily middle aged individuals (Galatro et al.) and the aged human microglia signature (present study). All three microglia signatures were established based on the same approach (fold change enrichment of microglia gene expression over the gene expression in their representative bulk tissue; parietal, temporal and dorsolateral cortex, respectively). The top 881 genes from each core signature were used for this comparison. (b) Pathway analysis of the microglia core genes unique to the current study (271 genes). Shown are Reactome entities that were significantly enriched in unique aged human microglia core genes. The orange diamonds represent the corrected enrichment p values for each Reactome entity (–log2(FDR)), while the black columns stand for the percentage of genes in any given Reactome entity that was covered by aged human microglia core genes. Abbreviations: FDR false discovery rate.

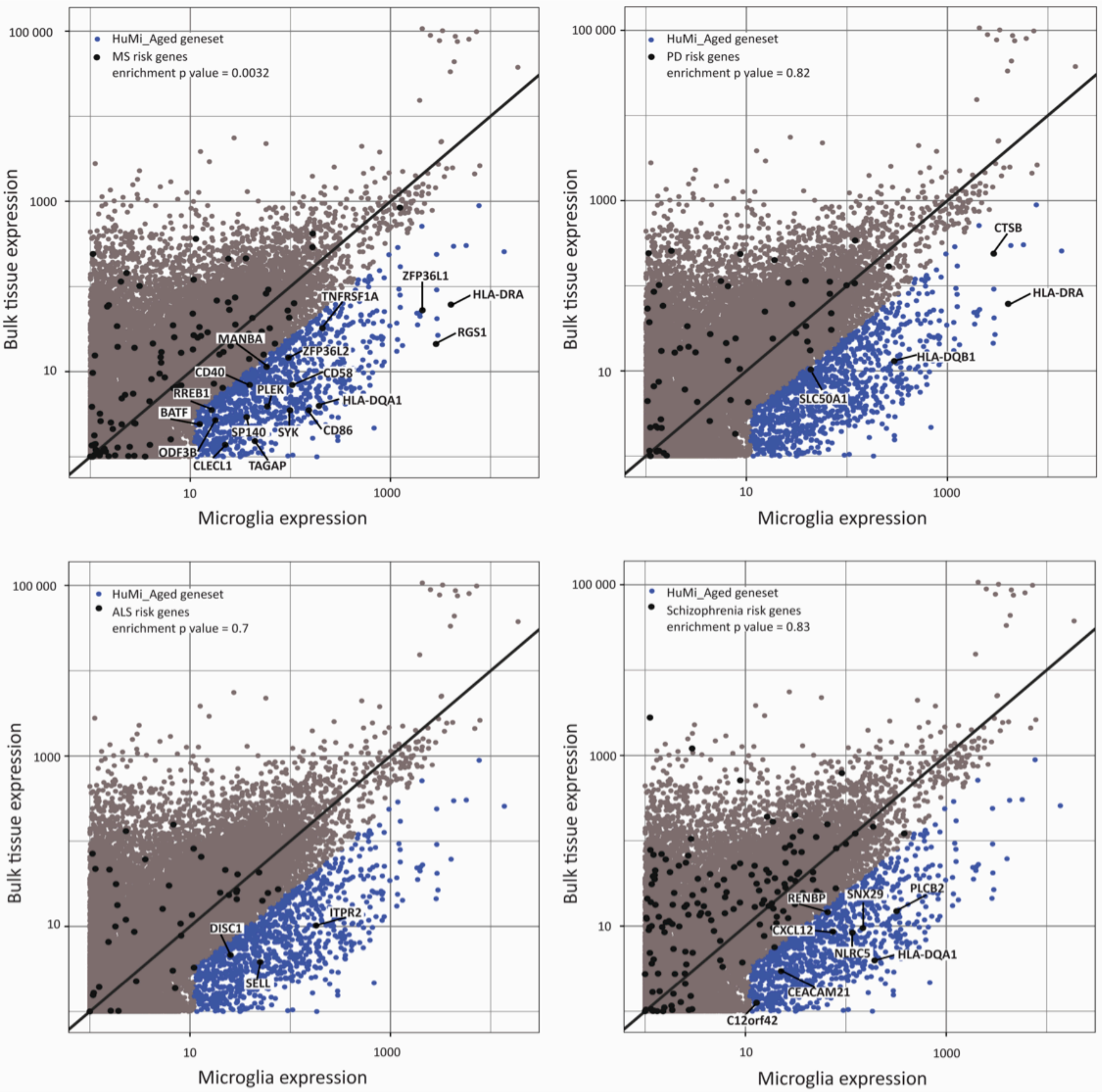


**Supplementary Figure 6. Relation of the HuMi_Aged to susceptibility genes.** Enrichment analysis of the microglia gene set (HuMi_Aged) in susceptibility genes for neurodegenerative and psychiatric diseases. Scatter plot depicting the distribution of gene expression values between the bulk DLPFC (N=540) and the isolated microglia (N=10). Each dot represents a gene. The X and the Y axes show normalized expression values. Blue dots represent the HuMi_Aged gene set. Single nucleotide polymorphisms that had previously been associated with neurodegenerative disease and psychiatric disorders were sourced from the NHGRI-EBI catalog of published genome-wide association studies. The enrichment of the HuMi_Aged gene set in the different disease associated genes was probed with an over-representation test. The HuMi_Aged geneset was found to be significantly enriched in MS risk genes, but not in genes involved in PD, ALS or schizophrenia. The

shared genes are represented by black dots in each scatter plot. Abbreviations: MS multiple sclerosis, PD Parkinson’s disease, ALS amylotrophic lateral sclerosis.

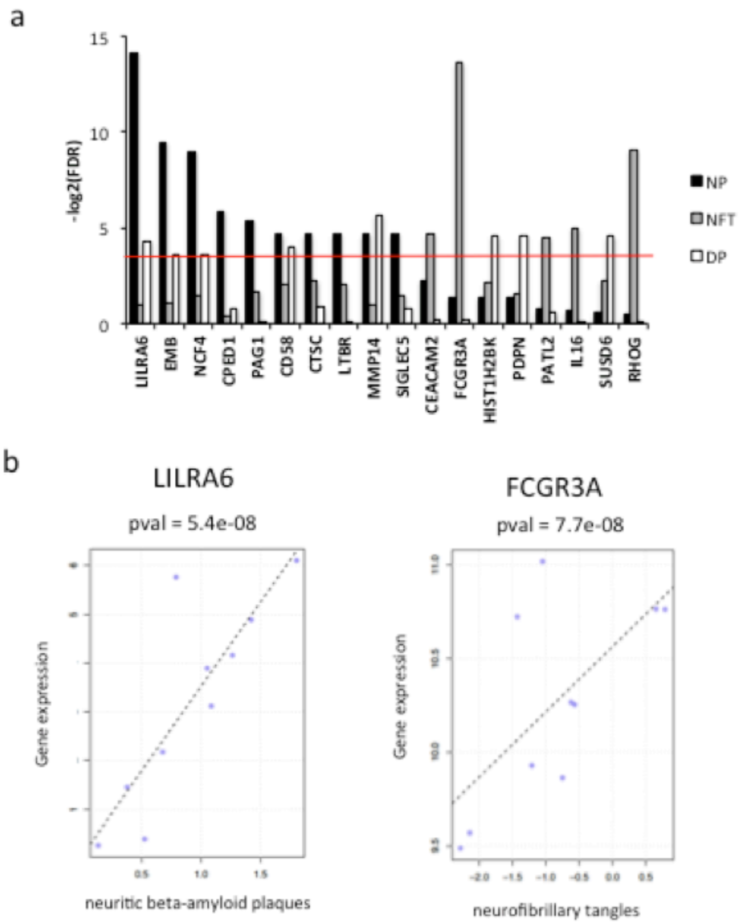


**Supplementary Figure 7. Trait associations in the microglia dataset.** The association of the gene expression with the neuropathological features of the aged brain was explored in the 10 microglia datasets. (a) Bar graph representing the genes that were significantly associated with at least one trait. (b) Representative scatter plots of the gene expression to trait associations for each neuropathological features. Abbreviations: NP neuritic plaques; NFT neurofibrillary tangles; DP diffuse plaques.

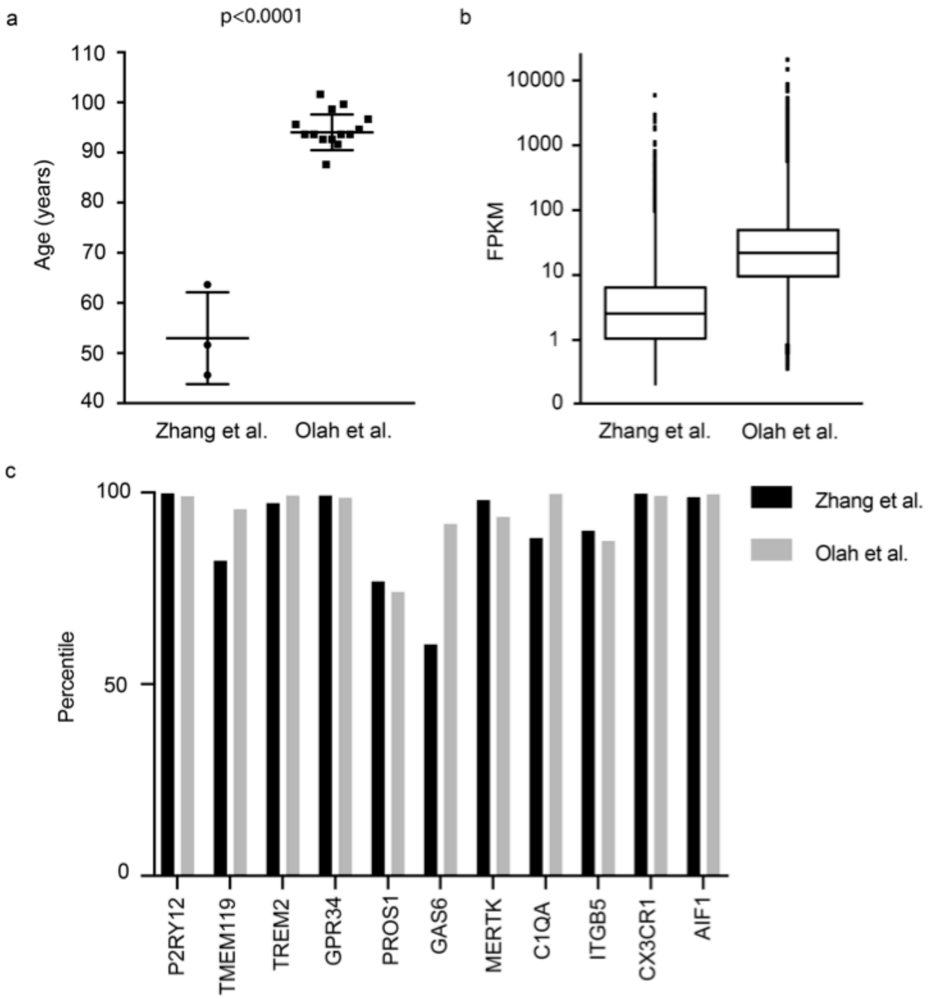


**Supplementary Figure 8. Human microglia datasets.** Comparison of middle-aged and aged adult human microglia datasets. (a) The donors contributing to the current study (N=10) were significantly older than the donors in another published transcriptomic study of adult human microglia (N=3) (Zhang et al.). Each individual donor is a symbol (circle or square) in the graph. (b) Boxplots depicting the per gene coverage in the Zhang et al. dataset compared to the dataset presented here. Only genes that had an FPKM value higher than 0.1 in both datasets were used for downstream analysis. Each dot in the graph is a gene. (c) Microglia signature genes were expressed at a comparable level in both the middle aged (Zhang et al.) and the aged (the present study) microglia transcriptomic datasets. Genes that have been shown to be only expressed in microglia when compared to monocytes (such as CX3CR1, P2RY12, TMEM119 and TREM2) ranked close to 100$^{th}$ percentile in both datasets, confirming the lack of substantial monocyte contamination. Abbreviations: FPKM Fragments Per Kilobase of transcript per Million mapped reads.

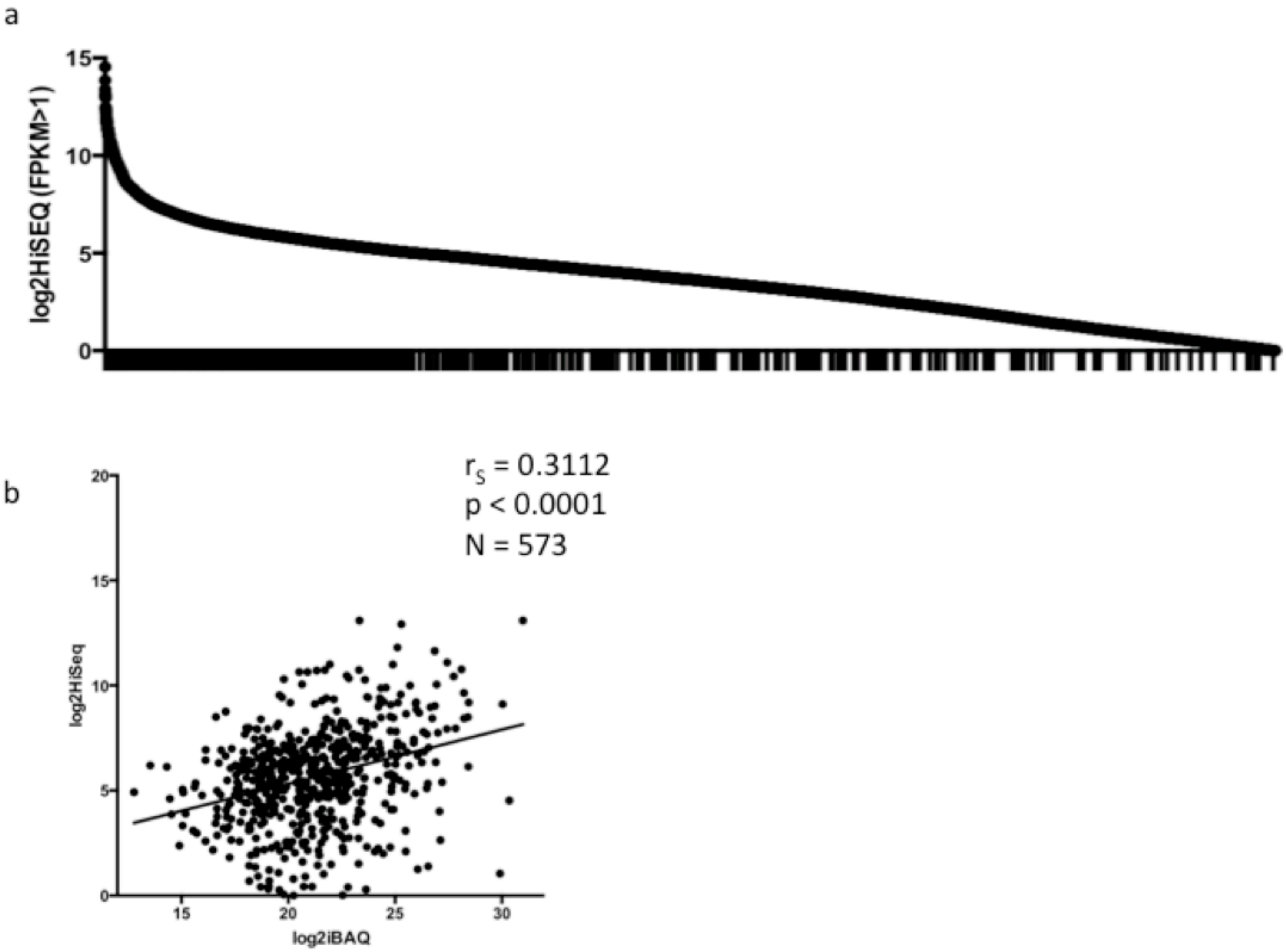


**Supplementary Figure 9. Shotgun proteomic profiling of human aged microglia.** LC-MS was used to investigate the proteome of aged human microglia. (a) The detection of proteins was slightly skewed towards the more highly expressed genes, but in accordance with the random nature of the shotgun approach, there was a reasonably good coverage of detection throughout the spectrum of gene expression levels. Genes were arranged in the order of their mean expression values in the RNA-Seq dataset. Each gene that was detected at the protein is represented by a vertical line on the x axis. For 75 detected proteins the FPKM value was below 1 in the RNA-Seq dataset (not shown). (b) Scatter plot showing the relationship between the protein and the RNA expression levels. Only genes and proteins that had a non-zero expression value were used.

**Supplementary Table 1. Clinicopathologic characteristics of the donors.**

| Donor No. | Study | cAD | Dementia | Braak stage | NIA Reagan | Global AD pathology burden | Amyloid | Tangles |
|---|---|---|---|---|---|---|---|---|
| 1 | MAP | 0 | 1 | 4 | 3 | 0.31 | 1.08 | 4.00 |
| 2 | MAP | 0 | 1 | 3 | 2 | 0.57 | 7.50 | 5.08 |
| 3 | ROS | 1 | 4 | 4 | 2 | 0.97 | 2.47 | 6.15 |
| 4 | MAP | 0 | 2 | 4 | 2 | 0.40 | 1.57 | 3.14 |
| 5 | MAP | 1 | 4 | 5 | 2 | 1.39 | 5.97 | 25.89 |
| 6 | MAP | 0 | 1 | 5 | 1 | 2.61 | 4.06 | 11.55 |
| 7 | MAP | 0 | 2 | 5 | 2 | 1.18 | 4.67 | 6.25 |
| 8 | MAP | 1 | 4 | 3 | 2 | 1.05 | 5.31 | 1.60 |
| 9 | ROS | 0 | 1 | 2 | 3 | 0.15 | 0.42 | 0.97 |
| 10 | MAP | 0 | 2 | 4 | 2 | 0.63 | 5.11 | 2.71 |

Study MAP – Memory and Aging Project; ROS – Religious Orders Study.

cAD Clinical diagnosis of Alzheimer's disease. Clinical diagnosis of Alzheimer's disease (AD) is based on criteria of the joint working group of the National Institute of Neurological and Communicative Disorders and Stroke and the Alzheimer's Disease and Related Disorders Association (NINCDS/ADRDA); the diagnosis of AD requires evidence of a meaningful decline in cognitive function relative to a previous level of performance with impairment in memory and at least one other area of cognition.

Dementia Clinical cognitive diagnosis summary. Six levels: 1 - NCI no cognitive impairment (CI); 2 - MCI mild cognitive impairment, no other condition contributing to CI; 3 - MCI+ mild cognitive impairment AND another condition contributing to CI; 4 - AD Alzheimer's disease dementia, no other condition contributing to CI (NINCDS/ADRDA Probable AD); 5 - AD+ Alzheimer's disease dementia AND other condition contributing to CI (NINCDS/ADRDA Possible AD); 6 - Other dementia, other primary cause of dementia, no clinical evidence of AD.

Braak stage

NIA Reagan NIA Reagan Diagnosis of Alzheimer's disease. Modified NIA-Reagan score based on consensus recommendations for postmortem diagnosis of Alzheimer's disease; the criteria rely on both neurofibrillary tangles (Braak) and neuritic plaques (CERAD); the criteria is modified because the neuropathological evaluation is done without knowledge of clinical information, including a diagnosis of dementia; the neuropathologist determines the level of AD pathology, those with intermediate or high fulfill criteria for having a pathologic diagnosis of AD; four levels: 1 - likelyhood of AD HIGH; 2 - likelyhood of AD INTERMEDIATE; 3 - likelyhood of AD LOW; 4 - no AD.

Global AD pathology burden A quantitative summary of AD pathology derived from counts of three AD pathologies (neuritic plaques, diffuse plaques and neurofibrillary tangles) in 5 different brain regions: midfrontal cortex, midtemporal cortex, inferior parietal cortex, entorhinal cortex and hippocampus.

Amyloid Overall amyloid levels. Amyloid beta protein was identified by immunohistochemistry using antibody against beta-amyloid 17-24 (clone 4G8) and quantified by image analysis; value is percentage of area occupied by amyloid; mean of 8 brain regions (angular gyrus, calcarine cortex, anterior cingulate cortex, entorhinal cortex, hippocampus, inferior temporal cortex, midfrontal cortex, superior frontal cortex).

Tangles Overall tangle density. Neuronal neurofibrillary tangles were identified by immunohistochemistry using antibodies against abnormally phosphorylated Tau protein (clone AT8); cortical density (per mm2) was determined using systematic sampling; mean of tangle score in 8 brain regions (angular gyrus, calcarine cortex, anterior cingulate cortex, entorhinal cortex, hippocampus, inferior temporal cortex, midfrontal cortex, superior frontal cortex).
