# Supplementary material for: A transcriptomic atlas of aged human microglia
Source: Nat Commun. 2018 Feb 7;9:539. doi: 10.1038/s41467-018-02926-5 (PMC5803269; doi:10.1038/s41467-018-02926-5)
Supplement: Supplementary file 2 — Description of Additional Supplementary Files [file 41467_2018_2926_MOESM2_ESM.pdf]

Description of Additional Supplementary Files

File Name: Supplementary Data 1
Description: The HuMi_Aged geneset. Identification of a microglia enriched gene set (HuMi_Aged) in the aged human brain. Genes that are preferentially expressed in microglia in the aged human brain were identified based on a four fold higher expression in the microglia RNA-Seq data (N=10) when compared to the bulk (dorsolateral prefrontal cortex, DLPFC) RNA-Seq expression (N=540).

File Name: Supplementary Data 2
Description: Meta-feature analysis of trait association. A meta-feature was constructed for the HuMi_Aged gene set. Subsequently we investigated the relationship of this meta-feature to the clinicopathological traits associated with aging and Alzheimer's disease in the bulk tissue level (DLPFC) data (N=540).

File Name: Supplementary Data 3
Description: Meta-feature analysis of trait association after stratification based on clinical AD diagnosis. A meta-feature was constructed for the HuMi_Aged gene set. Subsequently we investigated the relationship of this meta-feature to the clinicopathological traits associated with aging and Alzheimer's disease in the bulk tissue level (DLPFC) data (N=540) in donors with (cAD=1) and without (cAD=0) the clinical diagnosis of AD, separately.

File Name: Supplementary Data 4
Description: Differential gene expression analysis between middle aged and aged human microglia. The genes affected by aging were identified by contrasting the gene expression levels between midlle aged (microglia RNA-Seq dataset from Zhang et al.; mean age=53, SEM=±5.29, N=3) and aged human microglia (present study; mean age=94.07, SD=±0.95, N=10).

File Name: Supplementary Data 5
Description: The proteome of aged human microglia. A list of LC-MS detected microglia proteins and their relative abundance.
